# Zhentang Zhao: past and future of the Shanghai Synchrotron Radiation Facility

**DOI:** 10.1093/nsr/nwab185

**Published:** 2021-10-05

**Authors:** Weijie Zhao

**Affiliations:** NSR news editor based, Beijing

## Abstract

A synchrotron light source is familiar to many researchers. Biologists use it to determine structures of proteins and other biomacromolecules; material scientists use it to analyze internal structures of different materials; physicists use it to explore the basic matter states; and physicians use it to perform medical imaging. A high-performance synchrotron light source is often the workhorse supporting a prosperous multi-disciplinary research center. In China, that is the Shanghai Synchrotron Radiation Facility (SSRF).

Since its opening to users in 2009, SSRF has bred many significant scientific achievements and laid the foundation for the Shanghai Zhangjiang Cluster of Big Science Facilities. On 3 August 2021, *National Science Review* (*NSR*) interviewed Professor Zhentang Zhao, Director of the SSRF Science Center and vice president of the Shanghai Advanced Research Institute, Chinese Academy of Sciences (CAS). Prof. Zhao introduced the construction and development of the synchrotron light source and X-ray free electron laser facilities in Shanghai, as well as how SSRF experiences may be valuable for big science facilities and centers now under construction in China.

## THE PAST AND PRESENT OF SSRF

***NSR:*** Is SSRF the largest scientific facility in China?

***Zhao:*** It was the largest in 2009, when its construction was just completed. At that time, it was the most expensive and the largest big science facility in China. But now there are several larger ones, such as the Five-hundred-meter Aperture Spherical radio Telescope (FAST) and the China Spallation Neutron Source (CSNS). The Beijing High Energy Photon Source (HEPS), and the Shanghai High repetition rate XFEL and the extreme light facility (SHINE), which are under construction, are also larger.

However, SSRF is still the scientific facility with the largest number of users and scientific achievements in China. The construction and usage of SSRF accumulated some experience for later facilities in China.

***NSR:*** Would you summarize for us the construction process and the present status of SSRF?

***Zhao:*** SSRF is a medium-energy third-generation synchrotron light source. The third-generation synchrotron light sources can be classified into high-, medium- and low-energy ones according to the energy of their electron storage rings. The medium-energy ones circulate electrons at ∼3 GeV to generate light and have a relatively high cost-effective performance. They can generate high-quality light in the 20–30 keV region, where their performance can approach that of the high-energy ones. On the other hand, it can also generate high-brightness low-energy light in the 1 eV–1 keV range.

Currently there are about 20 medium-energy synchrotron light sources around the world, and SSRF is among the more advanced of them. Besides SSRF, DIAMOND in the UK, SOLEIL in France, ALBA in Spain and NSLS-II in the US are also medium-energy synchrotron light sources with very high performance.

**Figure 1:**
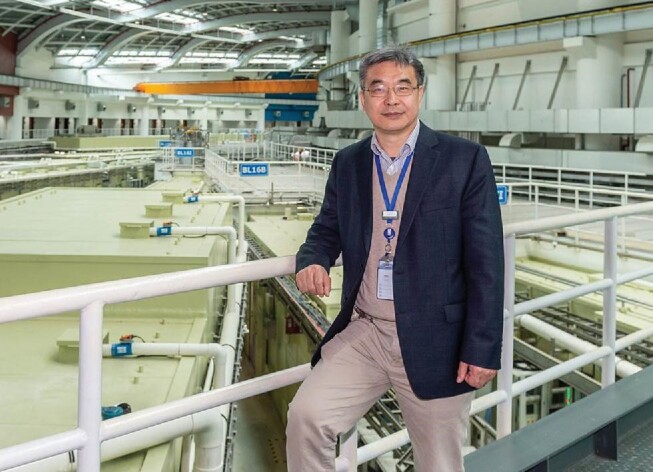
Prof. Zhentang Zhao in the experimental hall of SSRF (*courtesy of Prof. Zhentang Zhao).*

The construction of an advanced synchrotron light source in China was proposed as early as 1993 by three academicians of the Chinese Academy of Sciences (CAS), Shouxian Fang, Dazhao Ding and Dingchang Xian, based on international research trends and the research demand of Chinese scientists. This idea received immediate support from the CAS and the Shanghai Municipality. After more than 10 years of design and technical R&D, the construction of SSRF started on 25 December 2004, and the facility was officially opened for the first users on 6 May 2009.

Since then, SSRF has helped Chinese scientists solve a large number of scientific problems, and the facility has been extended continuously. In 2009, we had only seven beamlines. Now we have 23 beamlines and 34 experimental end stations in operation. Next year, in 2022, we will have 32 beamlines and 50 experimental end stations open for users. We have already built SSRF into a relatively mature world-class synchrotron radiation facility. The next 5 to 10 years will be the golden period of operations and scientific productivity at SSRF.

***NSR:*** Who are the users of SSRF? What are the major scientific achievements from SSRF?

***Zhao:*** Synchrotron light source produces electromagnetic radiations in the wavelength range of infrared to X-rays, which can help scientists to study molecular, electronic and magnetic structures of matter. It can be used for imaging and spectroscopic studies, as well as for structure determination, thus useful to both basic research and industrial applications. To date, SSRF has served more than 34 000 users from 3100 research groups throughout China. Among them, ∼50% were from universities, ∼30% from research institutions, ∼13% from high-tech companies and ∼7% from hospitals. Within the CAS system, researchers from more than 80% of all CAS institutions have made use of SSRF. In terms of discipline distribution, about a quarter of SSRF users are life scientists, and there are also many users from chemistry, materials science and energy sciences.

These users have conducted around 15 000 research projects at SSRF and made many important scientific findings. For example, Hong Ding's group from the CAS Institute of Physics experimentally discovered the Weyl fermion using SSRF. This is a breakthrough in physics and the publication was listed as one of the 49 selected papers for the 125th anniversary of the *Physical Review* journals. Another example is the structural screening of the novel anti-cancer drug Zanubrutinib, which is developed by China's biopharmaceutical company BeiGene and has been approved for clinical use in both China and the US. Our light source also helped determine the virus molecular structures of avian influenza viruses, Ebola virus, Zika virus and SARS-CoV-2, as well as the follow-up work on screening for anti-virus drugs and antibodies, and vaccine development. SSRF also helped scientists to solve their problems in many other areas, including efficient conversion of methane, single-atom catalysis, and the development of advanced materials such as super steel, graphene and carbon fiber. In the past 10 years, nine SSRF users’ research achievements appeared in the Top 10 Annual Scientific Advances in China, and five appeared in the Top 10 Annual Scientific and Technological News in China.

***NSR:*** Does international cooperation play a part in SSRF?

***Zhao:*** The development and construction of SSRF benefited greatly from international cooperation. During the early design and technical R&D period, we received a lot of help from facilities and institutions in the US, Japan and Europe. We adopted the standardized international review procedure for

To date, SSRF has served more than 34 000 users from 3100 research groups throughout China.

—Zhentang Zhao

the design and construction of SSRF. American scientists performed many rounds of reviews for us and helped us improve our design in many aspects. We also learned a lot from the user management and experimental proposal review procedures of the facilities in other countries, which helped us to build our own user management system.

Now, after many years of SSRF operations, we are in turn able to help other countries build their new light sources. In the upgrade of the ALS in the US, and the construction of new synchrotron light sources in South Korea and Brazil, we were able to offer some technological support, such as providing key components of sextupole magnets, in-vacuum undulators, or linac injectors. In the past, international communication mainly goes in one direction—we learned from our international peers. But now, with the development of China's science and technology (S&T), we are able to sit together with them to share our new technologies and discuss our common future directions.

Actually, in the past several decades, synchrotron radiation and particle accelerators are the research disciplines that did best in, and benefited most from international collaboration, which is the tradition of the high-energy physics field. Moreover, big facilities such as particle colliders and synchrotron light sources can act as centers for international cooperation, enabling scientists from different states and different disciplines to work together.

## FREE ELECTRON LASER FACILITIES

***NSR:*** The X-ray Free Electron Laser Test Facility (SXFEL-TF) passed national assessment at the end of 2020. The Shanghai High repetition rate XFEL and the extreme light facility (SHINE) is also under construction. What is the relationship among these three facilities?

***Zhao:*** These facilities are closely related. When designing SSRF, the free electron laser (FEL) facilities were already under consideration. In the late 1990s and early 2000s, Prof. Chen-Ning Yang wrote a series of nine letters to the leaders of the CAS and the Ministry of Science and Technology of China, as well as to the State Councilor for S&T, suggesting the development of a short wavelength FEL facility in China.

Before SXFEL-TF, we had been developing FEL technologies for more than 10 years. At the CAS Shanghai Institute of Applied Physics, we built the Shanghai Deep Ultraviolet Free Electron Laser (SDUV-FEL). On this platform, we mastered key technologies such as the photo-injector, low emittance linear accelerator, FEL undulator and beam modulation for producing FEL. We were also the first in the world in achieving the amplification of Echo-Enabled Harmonic Generation FEL. After SDUV-FEL, we also built the Extreme Ultraviolet FEL facility in the city of Dalian.

Based on these earlier studies and experiences, we completed the construction of SXFEL-TF in June 2020. It is a seeded FEL with a central wavelength of 8.8 nm. SXFEL-TF is located north of SSRF, with the closest part being only 50 meters away. In June 2021, we upgraded it into the Soft X-ray Free Electron Laser User Facility (SXFEL-UF), consisting of a 1.5 GeV linac, with a SASE FEL line, a seeded FEL line and five experiment stations. We have achieved SASE FEL lasing and saturation at the whole water window with a minimum wavelength of 2 nm, and investigated biological samples with the light. In 2022, we will open SXFEL-UF for the first users.

**Figure 2:**
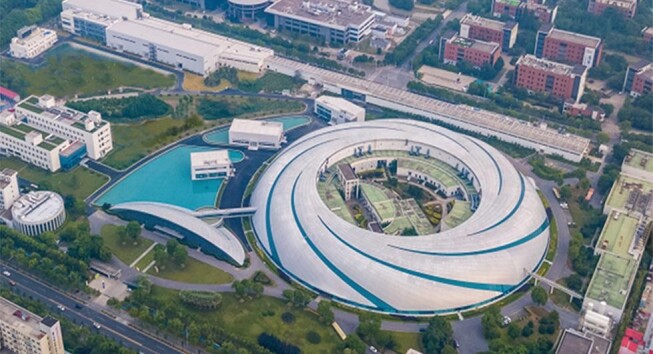
SSRF (the circular facility in the middle) and SXFEL (the linear facility on top) (*courtesy of Prof. Zhentang Zhao*).

Additionally, the construction of SHINE started in April 2018 and will be completed within several years. It is also a neighbor of SSRF. SHINE will be built in a 30-m-deep tunnel with a total length of 3.1 km, crossing the west part of the SSRF campus underground.

SHINE is a hard X-ray FEL facility based on an 8 GeV superconducting linac, covering photon energy from 0.4 keV to 25 keV. Its light pulse repetition rate can be as high as 1 MHz, which is 20 000 times higher than that of SXFEL-UF. Currently, there are only three hard X-ray FEL facilities of similar capability on the horizon. The European XFEL already in operation, and two under construction—LCLS-II in the US and SHINE in Shanghai.

In the future, we will combine the technologies of FEL and synchrotron radiation to upgrade SSRF into a coherent synchrotron light source, which can be recognized as a ‘fifth-generation light source’.

***NSR:*** What are the applications of FEL? Any difference from that of the synchrotron light source?

***Zhao:*** FEL is brighter than synchrotron light and has better coherence, and is thus capable of performing more precise imaging and structural analysis. Furthermore, the light pulse interval of FEL is 1000 times shorter than that of synchrotron light, so FEL can be used to study very fast processes, beyond the capability of synchrotron radiation. The synchrotron light source can take static images of molecules, while the FEL is able to take movies, capturing the fast dynamic processes of molecules or cells.

***NSR:*** FEL is much brighter, and its damage to the samples will be bigger. How can we solve this problem?

***Zhao:*** There are some solutions. For example, researchers are developing the technology of ‘detection before destruction’, in order to collect data in a very short time, and complete the measurement before the sample is damaged.

***NSR:*** Who will be the users of SXFEL and SHINE? Will it be similar to that of SSRF?

***Zhao:*** There will be some difference. In the first few years, the major users of FEL may be researchers in the basic sciences such as physics and chemistry, because FEL is able to help them solve some basic scientific problems. For example, using FEL, researchers in the US captured the dynamic breaking and forming process of chemical bonds, a feat unimaginable in the past. And of course, researchers of life sciences can use FEL to analyze the complexity and dynamics of biological processes that are relatively difficult for synchrotron radiation. There will also be laser science researchers using the brighter lights of FEL to study the scientific or technological issues of laser science itself.

Actually, at the early stage of SXFEL and SHINE construction, we sponsored a series of user meetings and workshops for domestic and international scientists to discuss the major scientific goals and applications of the FEL facilities in other countries, as well as the international need and expectation of our facility. Big science facilities should be guided by major scientific problems. This is the only way for our facility to be fully used and to make a bigger contribution to the development of S&T.

## THE ZHANGJIANG CLUSTER OF BIG SCIENCE FACILITIES

***NSR:*** Besides SSRF, SXFEL and SHINE, what are the other science facilities in the Zhangjiang Cluster of Big Science Facilities? What are the characteristics of this cluster?

***Zhao:*** This cluster is the basis of the Shanghai Zhangjiang Comprehensive National Science Center. Besides SSRF, SXFEL and SHINE, it also includes the Shanghai Superintense Ultrafast Laser Facility (SULF) and the Protein Science Facility (Shanghai). The latter includes equipment such as electron microscopes (EMs), nuclear magnetic resonance (NMR) spectrometers and mass spectrometers (MSs). In the future, we will also build an EM Center, an Ultrafast Electron Diffraction Facility and an Ultrafast Electron Microscopy Facility.

Compared with the other clusters of big science facilities in China, the internal link among the facilities in the Zhangjiang cluster is stronger. All facilities are based on photon science, producing light or using light, complementary in their capabilities, and can be co-utilized in some research projects. Internationally, more and more scientific centers are adopting this kind of combination. In Europe, Japan and the US, there are facility centers providing a synchrotron light source, FEL and EM.

***NSR:*** The Zhangjiang cluster is based on photon science. Will it promote research in photon science itself?

***Zhao:*** Yes. The key issue of photon science is how to efficiently generate and use light. The current major research goals are to increase the intensity and flux of the light sources, to make accelerator light sources smaller, to build accelerators with higher performance, to improve the temporal coherence of hard X-ray FELs, and to better detect and collect the fast signals generated by FEL. We will continuously study these issues and upgrade our facilities. That is how we can ensure our facilities are some of the best in the world.

## EXPERIENCES OF SSRF: HOW TO BUILD FUTURE LIGHT SOURCES?

***NSR:*** In China, new synchrotron light sources are being constructed in Beijing and Hefei. Are these synchrotron light sources complementary to each other?

***Zhao:*** The government has an overall plan on this issue. These synchrotron light sources are complementary in the energy range. As I said earlier, SSRF is a medium-energy light source at 3.5 GeV. Beijing is currently building a 6 GeV high-energy light source. Hefei has built a 0.8 GeV low-energy light source and is preparing to construct a 2.2 GeV low-energy one. These light sources have partially overlapping application scenarios, but also have their own characteristics. All three types of light sources are necessary for meeting the needs of S&T development.

Also, the demand from Chinese scientists for synchrotron light sources is very strong, and cannot be fulfilled by SSRF alone. Among all the SSRF user applications, less than 60% can pass our selection and receive some machine time, which is often only a quarter of the amount they requested. Among all the publications by Chinese scientists that made use of synchrotron light sources, about half had used light sources in other countries. It's obvious that SSRF is unable to fulfill all the demands.

***NSR:*** Is there demand for another medium-energy synchrotron light source in China?

***Zhao:*** It seems yes. At present, very strict selections of applications by expert groups are made in SSRF. Those focusing on major scientific problems are more likely to receive approval, whereas new projects with little preliminary results are often excluded. These new projects, especially those proposed by young researchers, need to be supported. Another medium-energy light source will help to meet this demand.

***NSR:*** Constructing a big science facility needs not only funding, but also experienced scientific teams. Do we have such teams for so many big facilities?

***Zhao:*** This is an important question. Many Chinese cities are now willing to invest land and money to build big science facilities, but our capacity to create construction teams is not big enough. If the experts are scattered to many facilities, it's very likely that no facility will be constructed to a high standard.

Thus, I think if China wants to build more light sources, we should not continue to use the construction approach of SSRF. We should not gather a big team for each facility and have that team perform all the design and construction work themselves. We may learn from the construction process of some light sources in other countries. We could transfer the technologies to a professional high-tech company, which could then undertake the task of constructing many devices.

## THE HAPPINESS OF LIGHT SOURCE CONSTRUCTORS

***NSR:*** You’ve been a light source designer, constructor and manager for more than 20 years. What are the moments of happiness in your work?

***Zhao:*** It was the moment when the light source emitted the first light beam and when it reached its designed performance. Before that moment, all the performance indexes are figures on a page, but at that moment, after such a long time of theoretical design, technological development and integrated debugging, when the blueprints finally became reality and yielded world-class performance, we were really happy and proud.

Another moment of happiness is when scientists, using our light source, solved long-puzzled-over problems, achieved major breakthroughs and received recognition from international peers. The scientific achievements belong to the scientists, but they are also reflections of the value of our light source, and the value of our years of hard work.

